# Importance of luminal membrane mesothelin expression in intraductal papillary mucinous neoplasms

**DOI:** 10.3892/ol.2015.2969

**Published:** 2015-02-17

**Authors:** TAKAHIRO EINAMA, HIROFUMI KAMACHI, HIROSHI NISHIHARA, SHIGENORI HOMMA, HIROMI KANNO, MARIN ISHIKAWA, FUTOSHI KAWAMATA, YUJI KONISHI, MASANORI SATO, MUNENORI TAHARA, KUNIAKI OKADA, SHUNJI MURAOKA, TOSHIYA KAMIYAMA, AKINOBU TAKETOMI, YOSHIHIRO MATSUNO, HIROYUKI FURUKAWA, SATORU TODO

**Affiliations:** 1 Department of General Surgery, Hokkaido University Graduate School of Medicine, Sapporo, Hokkaido 060-8638, Japan; 2 Department of Surgery, Hokkaido Social Work Association Obihiro Hospital, Obihiro, Hokkaido 080-0805, Japan; 3 Division of Gastroenterological and General Surgery, Asahikawa Medical University, Asahikawa, Hokkaido 078-8510, Japan; 4 Department of Translational Pathology, Hokkaido University Graduate School of Medicine, Sapporo, Hokkaido 060-8638, Japan; 5 Department of Pathology, Laboratory of Cancer Research, Hokkaido University School of Medicine, Sapporo, Hokkaido 060-8638, Japan; 6 Department of Surgery, JA Sapporo Kosei Hospital, Sapporo, Hokkaido 060-0033, Japan; 7 Department of Pathology, JA Sapporo Kosei Hospital, Sapporo, Hokkaido 060-0033, Japan; 8 Department of Surgical Pathology, Hokkaido University Hospital, Sapporo, Hokkaido 060-8638, Japan

**Keywords:** mesothelin, intraductal papillary mucinous neoplasms, luminal membrane expression

## Abstract

The present study demonstrated that luminal membrane mesothelin expression is a reliable prognostic factor in gastric cancer. Intraductal papillary mucinous neoplasms (IPMNs) often exhibit a spectrum of dysplasia, ranging between adenoma and carcinoma. Therefore, an immunohistochemical analysis of mesothelin expression in IPMN was performed in the present study, focusing on the localization of mesothelin. IPMNs were classified into two groups, IPMNs associated with invasive carcinoma and low-high (L-H) grade dysplasias. The tumors were classified as mesothelin-positive or -negative and in the mesothelin-positive cases, the localization of mesothelin was evaluated as luminal membrane- or cytoplasmic-positive. Among the 37 IPMNs, mesothelin expression was observed in 21 samples (56.8%), including 46.2% (12 out of 26) of the L-H dysplasia and 81.8% (9 out of 11) of the invasive carcinoma samples (P=0.071). Luminal membrane localization was observed in 10 samples (27%), including 15.4% (4/26) of the L-H dysplasia samples and 54.5% (6 out of 11) of the invasive carcinoma samples (P=0.022). Six patients experienced post-operative recurrence, with five of the recurrent tumors exhibiting mesothelin expression and all six exhibiting luminal membrane localization. It was concluded that immunohistochemical examinations for mesothelin expression and localization are clinically useful for prognostic assessments and decision making regarding further treatment subsequent to surgical procedures in patients with IPMN.

## Introduction

Mesothelin is a 40-kDa cell-surface glycoprotein that is expressed in the normal mesothelial cells lining the pleura, pericardium and peritoneum (1–2). Overexpression of mesothelin has also been identified in several types of cancer, including mesothelioma, ovarian cancer and pancreatic cancer (3–6). The full-length human mesothelin gene codes for a 71-kDa precursor protein, which is cleaved by furin-like proteases into a 40-kDa C-terminal fragment that remains membrane bound and a 31-kDa N-terminal fragment, which is secreted into the blood. The C-terminal 40-kDa fragment is termed mesothelin and is attached to the cell membrane via a glycosyl-phosphatidylinositol (GPI) anchor (1).

The biological function of mesothelin is not fully understood, although previous studies have suggested that mesothelin overexpression increases cell proliferation and migration (7). In pancreatic cancer, a previous study found that the expression of mesothelin is associated with unfavorable outcomes (8). Furthermore, the localization of mesothelin in gastric cancer, extrahepatic bile duct cancer and colorectal adenocarcinoma was also investigated in each study. It was found that the expression of mesothelin at the luminal membrane was a reliable prognostic factor, suggesting that membrane-localized mesothelin plays a functionally significant role in promoting aggressive behavior in the aforementioned cancers (9–11).

Intraductal papillary mucinous neoplasm (IPMN) often exhibits a spectrum of dysplasias, ranging between adenoma and carcinoma in the same lesion (12). To date, however, there have not been any studies regarding the significance of mesothelin expression in IPMN. Therefore, an immunohistochemical analysis of mesothelin expression in IPMN was performed in the present study, focusing on the localization of mesothelin, determining whether mesothelin is present in the luminal membrane or cytoplasm.

## Materials and methods

### Patients and tumor specimens

The present study was performed with the approval of the Internal Review Board on Ethical Issues of Hokkaido University Hospital (Sapporo, Hokkaido, Japan), and written informed consent was obtained from the patients. The subjects consisted of 37 patients who underwent surgery with curative intent for IPMN between January 2000 and December 2006 at the Department of General Surgery (Hokkaido University, Graduate School of Medicine, Sapporo, Japan) or JA Sapporo Kosei Hospital (Sapporo, Japan). The IPMNs were classified into two groups, IPMNs associated with invasive carcinoma, termed invasive carcinomas, and those associated with low to high (L-H) grade dysplasias, termed L-H dysplasias, according to the 2010 World Health Organization criteria (12). The clinicopathological characteristics of these cases are summarized in Table I.

Out of the 37 patients with IPMN, 26 (70.3%) were classified as possessing L-H grade dysplasia and the remaining 11 patients (39.7%) were categorized as possessing invasive carcinoma. The mean age of the cohort was 67.2 years (standard deviation, ±9.7 years). In total, 24 patients (64.9%) were male and the remaining 13 patients (35.1%) were female. The tumors were classified as branch duct type tumors in 25 cases (67.6%), main duct tumors in 10 cases (27.0%), and combined type tumors in two cases (5.4%). Mural nodules were identified in 31 patients (83.8%). Of the 37 patients, four succumbed to the disease, and the median follow-up period of the surviving 33 patients was 50.4 months (range, 5.9–103.0 months).

Formalin-fixed paraffin-embedded tissue blocks were prepared from the tumor specimens. The sections were then cut and stained using hematoxylin and eosin, prior to being used for routine histopathological examinations. All the tumors were diagnosed as IPMN. A representative tissue block was selected from each case and used for the immunohistochemical examinations.

### Immunohistochemistry

The immunohistochemical staining of mesothelin was performed as previously described (8). Tissue sections (4-μm thick) were mounted on charged glass slides, deparaffinized and rehydrated through a graded ethanol series. For antigen retrieval, Dako Target Retrieval Solution (pH 9.0; catalogue number, S2368; Dako Denmark A/S, Glostrup, Denmark) was used, and the slides were boiled in a pressure cooker (Pascal Pressure Cooker; model, S2800; Dako North America, Inc., Carpinteria, CA, USA) at 125°C for 3 min. The sections were treated with 0.3% hydrogen peroxidase for 5 min to quench endogenous peroxidase activity. Subsequently, the slides were incubated with a 1:50 dilution of a mouse monoclonal antibody for mesothelin (clone 5B2; Novocastra, Newcastle-Upon-Tyne, United Kingdom) at room temperature for 30 min. The slides were then reacted with a dextran polymer reagent combined with horseradish peroxidase-conjugated secondary antibodies (Envision/HRP; Dako North America, Inc.) for 30 min at room temperature. Specific antigen-antibody reactions were visualized using 0.2% diaminobenzine tetrahydrochloride and hydrogen peroxide. The slides were counterstained with hematoxylin for 10 min and then rinsed gently in reagent quality water.

### Immunohistochemical evaluation

All assessments concentrated on the tumor-bearing regions of the specimens. Each slide was evaluated independently by three pathologists who were unaware of the clinical outcomes.

The immunostaining of mesothelin was evaluated in terms of the proportion of stained tumor cells and the staining intensity in each case. The proportion of immunostained mesothelin-positive cells was assessed as follows: +1, 1–10% of cells were stained; +2, 10–50% of cells were stained; and +3, >50% of cells were stained. The mesothelin staining intensity was evaluated as weak (+1) or moderate to strong (+2) and the localization of the staining was recorded as luminal membrane or cytoplasmic. The final mesothelin expression results for each case were then determined using the following scoring system, which was developed in a previous study of pancreatic cancer (8): mesothelin-positive was defined as a proportion score of ≥+3 and an intensity score of +2, while mesothelin-negative was defined as a total score of <+3, except in cases involving a proportion score of +1 and an intensity score of +2.

**Figure 1:**
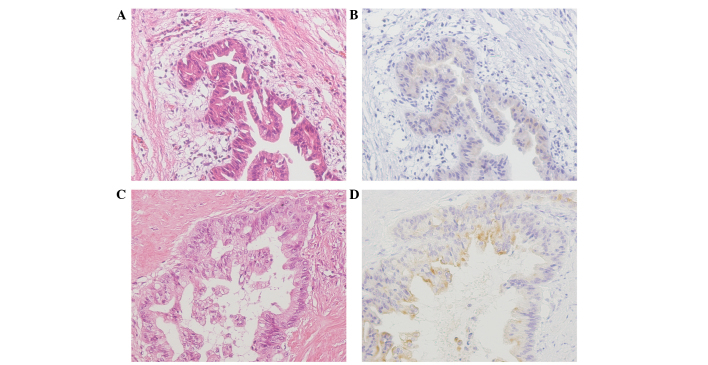
Representative samples of pancreatic ductal adenocarcinoma tissues that were awarded mesothelin expression scores of 0, +1, and +2. (A) Intraductal papillary mucinous adenoma tissue sample (stain, HE). (B) Mesothelin expression was faintly or barely detected in the tumor cell cytoplasm of (A) (mesothelin staining intensity of +1). No luminal membrane staining was detected in the adenoma cells. (C) Invasive intraductal papillary mucinous carcinoma (stain, HE). (D) Moderate to strong mesothelin expression was detected in the carcinoma cells (mesothelin staining intensity of +2). Granular cytoplasmic staining and staining of the entire cell membrane circumference were detected. This case was classified as luminal membrane-positive and cytoplasm-positive. Magnification, ×200. HE, hematoxylin and eosin.

Furthermore, among the mesothelin-positive cases, the localization of mesothelin was evaluated as luminal membrane or cytoplasmic. Cases in which the entire circumference of the luminal membrane was clearly stained throughout the section were defined as luminal membrane-positive. Conversely, cases in which the luminal membrane was stained discontinuously or faintly, or cases in which no luminal membrane staining was observed, were defined as luminal membrane-negative. Cytoplasmic mesothelin expression was evaluated and cases in which cytoplasmic staining was clearly observed in the constituent cancer cells, including cytoplasmic granular staining, were defined as cytoplasm-positive (Fig. 1) (9).

### Statistical analysis

The χ^2^-squared test or Fisher’s exact test were used to determine the correlations between the mesothelin expression results and each clinicopathological parameter. All differences were considered significant at P<0.05. All statistical analyses were performed using StatView 5.0 software (SAS Institute Inc., Cary, NC, USA).

## Results

### Mesothelin expression was detected in IPMN tissue, but not in the normal pancreatic tissue

**Figure 2:**
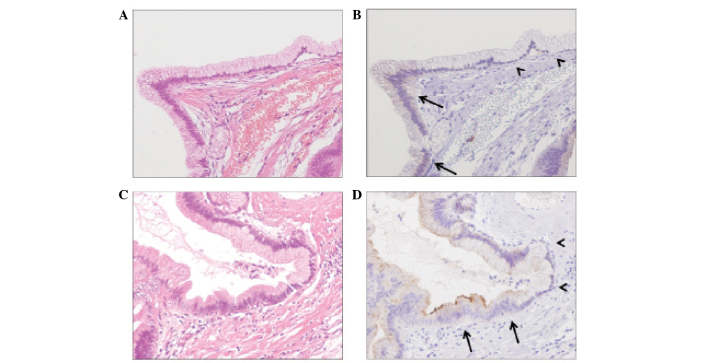
Mesothelin expression was detected in IPMN tissue, but not in the normal pancreatic tissue, and was limited to adenoma cells (arrows) Notably, the benign ductal epithelial cells of the intraductal papillary mucinous adenoma patients were negative for mesothelin expression (arrowheads). (A) Stain, hematoxylin and eosin (HE). (B) Tissue sample in (A) stained for mesothelin expression. (C) Stain, HE. (D) Tissue sample in (C) stained for mesothelin expression. Magnification, ×200.

All the benign pancreatic tissues were negative for mesothelin expression. Conversely, mesothelin expression was detected in adenoma and carcinoma cells. The majority of the adenoma cells that expressed mesothelin exhibited slight diffuse cytoplasmic staining (Fig. 2).

### Recurrence of IPMN

The recurrence of IPMN was detected in six cases. The recurrence was located in the lymph nodes in two patients and in the peritoneum, liver and pleura in one patient each.

### Mesothelin expression in IPMN

The findings of the present study regarding mesothelin expression are summarized in Table II. Among the 37 cases of IPMN, mesothelin expression was observed in 46.2% (12 out of 26) of the samples from L-H grade dysplasia and 81.8% (nine out of 11) of the samples from invasive carcinomas. Luminal membrane mesothelin expression was observed in 15.4% (four out of 26) of the L-H grade dysplasia samples and 54.5% (six out of 11) of the invasive carcinoma samples. Cytoplasmic mesothelin expression was observed in 38.5% (10 out of 26) of the L-H grade dysplasias and 63.6% (seven out of 11) of the invasive carcinomas. The incidence of luminal membrane mesothelin expression was correlated with the histological classification of the tumor (P=0.022) and the recurrence rate (P=0.0030). There were no significant correlations between the histological classification and any of the other clinicopathological parameters (Table III).

### The association between the mesothelin expression and recurrence of IPMN

Among the 37 IPMN patients, six suffered post-operative recurrence. In five of the cases with recurrent tumors, the tumors exhibited mesothelin expression, and all five tumors exhibited luminal membrane mesothelin expression (Table IV).

## Discussion

In the present study, it was demonstrated that luminal membrane mesothelin expression in IPMN is associated with poor post-operative clinical outcomes. These results support the findings of previous studies investigating mesothelin expression in gastric cancer, extrahepatic bile duct cancer, and colorectal adenocarcinoma (9–11).

The possible mechanism responsible for the membranous localization of mesothelin may be based on the full-length human mesothelin gene encoding a 71-kDa precursor protein. This protein is proteolytically cleaved by furin-like proteases into an N-terminal secreted form and a C-terminal fragment, 40-kDa mesothelin, which is a GPI-linked glycoprotein (1,13,14). The 5B2 anti-mesothelin antibody, which was employed in the immunohistochemical examination in the present study, is able to detect the 71-kDa precursor protein and the 40-kDa C-terminal fragment, but not the 30-kDa N-terminal fragment. Thus, based on the reported molecular processing mechanism of mesothelin and the specificity of the 5B2 antibody, the luminal membrane staining observed in the present study is likely to have indicated the presence of the 40-kDa membrane-bound form of mesothelin, while the cytoplasmic staining is likely to have indicated the presence of the 71-kDa precursor form of mesothelin. The present results are consistent with the results from previous studies, and support the hypothesis that the 40-kDa membrane-bound form of mesothelin is the active form, which promotes aggressive cellular characteristics, including an increase in cell motility, invasive or migratory ability, and growth of metastatic tumors (15–17). In addition, Kawamata *et al* demonstrated that the biological function of 40-kDa mesothelin is associated with lymphatic cancer cell invasion *in vitro* (10).

**Figure 3:**
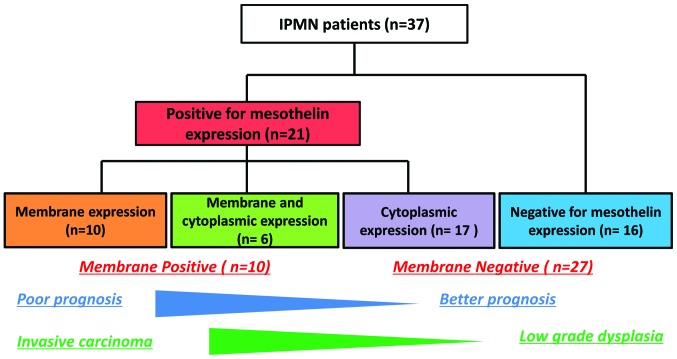
Flow chart of mesothelin expression in IPMN cells. IPMN, intraductal papillary mucinous neoplasm.

Pancreatic IPMNs exhibit a histological spectrum ranging between benign adenoma and invasive cancer (12). The cyst diameter, main pancreatic duct-type lesions and the presence of mural nodules are associated with histologically malignant grades of IMPN, and these criteria are widely used to exclude benign lesions from surgical intervention (18–20). At present, large numbers of benign lesions undergo surgical resection, which is suboptimal. As accurate pre-operative imaging-based assessments of malignancy are not currently possible, a method for identifying pre-invasive lesions and the establishment of a novel molecular-based management strategy is required. Appropriate criteria that can be used to identify IPMN containing rapidly invasive adenocarcinoma components are required. This would allow less aggressive lesions to simply be followed-up and avoid unnecessary surgery. Immunohistochemical evaluations of luminal membrane mesothelin expression in IPMN are considered to be of clinical benefit as they provide prognostic information (Fig. 3).

In conclusion, the present study demonstrated the prognostic significance of luminal membrane mesothelin expression in IPMN, although additional studies involving an increased number of luminal membrane-positive cases are required to confirm the present findings. Immunohistochemical examination of mesothelin expression in surgically resected tumor specimens is clinically useful for assessing the prognosis and for deciding on the necessity of additional treatment following surgical procedures in patients with IPMN.

## Figures and Tables

**Table I:** Clinicopathological characteristics of the 37 IPMN patients.

		Group		
			
Parameter	Total (n=37)	Low-high dysplasia (n=26)	Invasive carcinoma (n=11)	P-value
Age, years ± SD	67.2±9.7	65.7±9.7	64.3±11.0	0.71
Gender, n				
Male	24	14	10	0.057
Female	13	12	1	
Type of IPMN, n				
Main duct or branch	12	11	1	0.064
Combined	25	15	10	
Mural nodules, n (%)	31 (83.8)	20 (76.9)	11 (100.0)	0.15
Recurrence, n				
Yes	6	0	6	0.0002
No	31	26	5	

SD, standard deviation; IPMN, intraductal papillary mucinous neoplasm.

**Table II:** Associations between the expression pattern of mesothelin and clinicopathological parameters.

		Mesothelin expression			Luminal membrane expression			Cytoplasmic expression		
							
Factors	n	Positive (n=21)	Negative (n=16)	P-value	Positive (n=10)	Negative (n=27)	P-value	Positive (n=17)	Negative (n=20)	P-value
Histological classification, n										
Low-high dysplasia	26	12	14	0.071	4	22	0.022	10	16	0.28
Invasive carcinoma	11	9	2		6	5		7	4	
Type of IPMN, n										
Main or combined	12	4	8	0.077	1	11	0.12	4	8	0.32
Branch	25	17	8		11	16		13	12	
Mural nodules, n										
Yes	31	18	13	0.99	10	21	0.16	15	16	0.67
No	6	3	3		0	6		2	4	
Tumor diameter, n										
≥3 cm	25	15	10	0.73	8	17	0.44	12	13	0.99
<3 cm	12	6	6		2	10		5	7	
Recurrence, n										
Yes	6	5	1	0.21	5	1	0.0030	3	3	0.18
No	31	16	15		5	26		14	17	

IPMN, intraductal papillary mucinous neoplasm.

**Table III:** Association between histological classification and clinicopathological parameters.

		Group		
			
Factors	Total (n=37)	Low-high dysplasia (n=26)	Invasive carcinoma (n=11)	P-value
Type of IPMN, n				
Main or combined	12	11	1	0.064
Branch	25	15	10	
Mural nodules, n				
Yes	31	20	11	0.15
No	6	6	0	
Tumor diameter, n				
≥3 cm	25	16	9	0.28
<3 cm	12	10	2	
Mesothelin membrane expression, n				
Yes	10	4	6	0.022
No	27	22	5	

IPMN, intraductal papillary mucinous neoplasm.

**Table IV:** Mesothelin expression in patients with recurrent disease.

Case	Age, years	Gender	Mesothelin expression	Mesothelin localization	

				Membrane	Cytoplasm
1	80	M	+	+	+
2	80	M	+	+	−
3	81	M	+	+	+
4	55	M	+	+	+
5	66	M	+	+	+
6	72	F	−	−	−

M, male; F, female; +, positive; −, negative.
